# Effect of Wide-Spectrum Monochromatic Lights on Growth, Phytochemistry, Nutraceuticals, and Antioxidant Potential of In Vitro Callus Cultures of *Moringa oleifera*

**DOI:** 10.3390/molecules28031497

**Published:** 2023-02-03

**Authors:** Muhammad Naeem Bajwa, Mehnaz Khanum, Gouhar Zaman, Muhammad Asad Ullah, Umar Farooq, Muhammad Waqas, Nisar Ahmad, Christophe Hano, Bilal Haider Abbasi

**Affiliations:** 1 Department of Biotechnology, Quaid-i-Azam University, Islamabad 45320, Pakistan; 2 School of Agriculture and Food Sciences, Gatton Campus, The University of Queensland, Gatton, QLD 4343, Australia; 3 Center for Biotechnology and Microbiology (CB&M), University of Swat, Swat 19200, Pakistan; 4 Laboratoire de Biologie des Ligneux et des Grandes Cultures (LBLGC), University of Orleans, INRAE USC1328, F28000 Chartres, France; 5 Pakistan Academy of Sciences, Islamabad 44000, Pakistan

**Keywords:** *Moringa oleifera*, phytochemistry, elicitation, phytochemicals, antioxidant, nutraceutical, monochromatic light

## Abstract

*Moringa oleifera*, also called miracle tree, is a pharmaceutically important plant with a multitude of nutritional, medicinal, and therapeutic attributes. In the current study, an in-vitro-based elicitation approach was used to enhance the commercially viable bioactive compounds in an in vitro callus culture of *M. oleifera*. The callus culture was established and exposed to different monochromatic lights to assess the potentially interactive effects on biomass productions, biosynthesis of pharmaceutically valuable secondary metabolites, and antioxidant activity. Optimum biomass production (16.7 g/L dry weight), total phenolic contents (TPC: 18.03 mg/g), and flavonoid contents (TFC: 15.02 mg/g) were recorded in callus cultures placed under continuous white light (24 h), and of other light treatments. The highest antioxidant activity, i.e., ABTS (550.69 TEAC µM) and FRAP (365.37 TEAC µM), were also noted under white light (24 h). The analysis of phytochemicals confirmed the significant impact of white light exposures on the enhanced biosynthesis of plant secondary metabolites. The enhanced levels of secondary metabolites, i.e., kaempferol (1016.04 µg/g DW), neochlorogenic acid (998.38 µg/g DW), quercetin (959.92 µg/g DW), and minor compounds including luteolin, apigenin, and p-coumaric acid were observed as being highest in continuous white light (24 h with respect to the control (photoperiod). Similarly, blue light enhanced the chlorogenic acid accumulation. This study shows that differential spectral lights demonstrate a good approach for the enhancement of nutraceuticals along with novel pharmacologically important metabolites and antioxidants in the in vitro callus culture of *M. oleifera*.

## 1. Introduction

To achieve sustainable development, global climate change, the limited supply of food, restricted supply of fresh water, and increasing energy consumption and demand are amongst the most critical global challenges currently faced by humanity [1]. The search and need for plants and herbal products that can meet rising food and medicine demands in the circumstances of climate change and food scarcity is a serious global challenge nowadays, particularly in areas where malnutrition persists [2]. Plants are endorsed as an enriched reservoir of important pharmaceutical products due to ample therapeutic facets and have been used in traditional therapeutic herbal medicines against numerous diseases and illnesses. For millennia, medicinal plants have formed the backbone of traditional medicines and are regarded as a potential source of biologically active compounds of therapeutic value. It has been estimated by the World Health Organization that more than 80% of the population depends on traditional phytomedicines for some aspects of their primary health care requirements. Primary and secondary metabolites present in plants are the major source of economically important commercial products like food additives, fragrances, pesticides, herbal cosmetics, dyes, flavors, pharmaceuticals, pesticides, essential oil, natural rubber, gum, waxes, tannins. The global herbal industry shares about US$100 billion and is forecasted to expand at a faster rate between 2020 and 2030 [1,3]. The in vitro growth of plant cells in a controlled environment is a highly accepted, eco-friendly method for the long-term supply of valuable phytochemicals, even from rare, protected, endemic, threatened, or endangered plant species. The pharmaceutical industry is diverting towards medicinal plants with the latest biotechnological approaches for the enhanced biosynthesis of some distinct novel compounds having a specific therapeutic and metabolic role [3]. *Moringa oleifera*, also called as the miracle tree, ben oil, drumstick, and horseradish tree, from genus *moringacea*, is a plant that has multiples purposes and is a complete source of food and diet components; its leaves, pods, and roots are used as both food and medicine [4,5]. It has very high nutritional value, containing carbohydrates, proteins, lipids, essential amino acids and minerals, vitamins, β-carotenoid along with phenolics, terpenoids, and steroids [6]. *M. oleifera* leaf extract has previously been reported as anti-hypertensive [7], cardio-protective [8], anti-diabetic or hypoglycemic in rats [9], antioxidant [10], anti-inflammatory [11], anti-urolithiatic [12], anthelmintic [13], anti-ulcer [14], analgesic [15], anti-bacterial [16], and anti-viral [17], due to the presence of diverse metabolites. *M. oleifera* is a plant recognized as the best alternative for the prevention and alleviation of stunted growth and malnutrition challenges [18].

Innovative approaches in biotechnology are corroborating the significance of in vitro plant tissue culture techniques in plant breeding and genetics for either biosynthesis of pharmaceutically novel metabolites or a model system to study plant physiology, biochemistry, and pathology [19,20]. The production of such novel metabolites in concentrated form through tissue culture techniques has been reported in the literature [21,22]. Phenolics and flavonoids are the high valued plant secondary metabolites (SM) present in medicinal plants. They serve as a defense mechanism and provide protection against several reactive oxygen species produced during stress conditions and exhibit significant therapeutic potential against a variety of diseases [23]. These precious metabolites can be isolated from naturally available plants grown in the wild, but their commercial production is limited because of various ecological, regional, and seasonal constraints. Moreover, due to deforestation, overexploitation, and pollution, it is often not convenient to obtain extracts or secondary metabolites directly from wild plants. The plant tissue culture (PTC) technique presents an alternate platform to overcome such limitations. This technique can be used for the large-scale production of high-value secondary metabolites in in vitro cell cultures [24]. Plant tissue culture techniques are useful for the efficient biosynthesis of commercially and medicinally important bioactive compounds within a short period of time. Callus cultures are considered as potential biofactories that provide a steady manufacturing system and safeguard the sustainable supply of secondary metabolite products without destroying the plants’ natural habitat [25].

Elicitation is one of the best approaches for instigating the defense response in plants for the induction of chemical responses against particular stress. The type, dose, nature, and exposure time of an elicitor are required for provoking strong elicitation effects in in vitro callus cultures [23]. Amongst the various elicitation approaches, light as an elicitor is widely praised in the literature for provoking increased biosynthesis of secondary metabolites of various plants that have medicinal importance through in vitro productions [26]. Shama et al. and Turtoi intensively studied the useful repercussion of light including restrained chlorophyll degradation, combating pathogens, and overall effect on nutritional factors [27,28]. Through recent developmental approaches, the use of elicitors has proven to be an efficient system for the synthesis of biomass and secondary metabolites [29]. No study—to the best of our knowledge—in the literature has discussed the pattern in metabolites production/accumulation under differential light exposure in *M. oleifera* in vitro cultures. Differential lights affect plants differently based on their inherent genetic, morphological, and physiological framework. *M. oleifera* is a quite unique plant with a range of medicinal and nutritional benefits (as discussed previously). Therefore, an in vitro setup was designed to quantitatively determine the effects of differential lights on overall biomass production and medicinally important metabolite accumulation.

## 2. Results and Discussion

### 2.1. Trend in Biomass Accumulation

**Figure 1:**
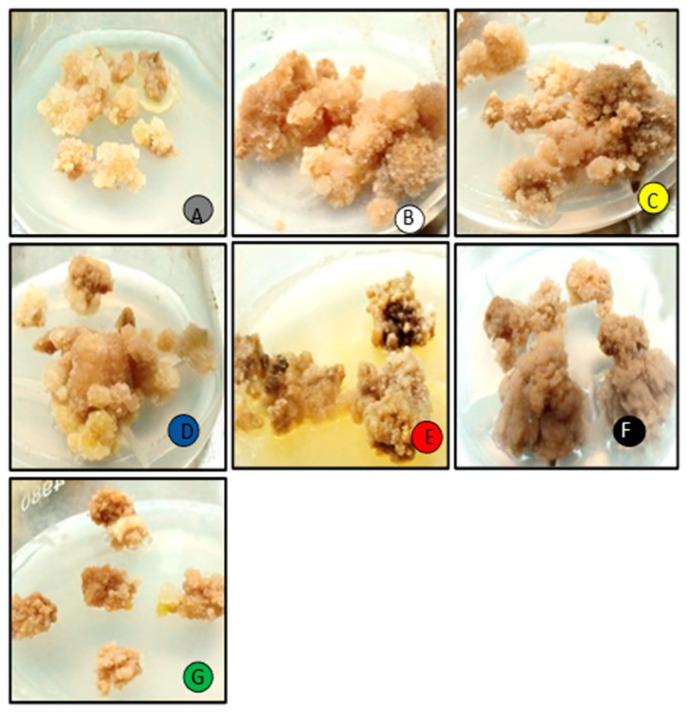
The morphological changes in the callus culture of *M. oleifera* kept under different monochromatic lights. ((**A**) = Control; (**B**) = White; (**C**) = Yellow; (**D**) = Blue; (**E**) = Red; (**F**) = Dark; (**G**) = Green)).

**Figure 2:**
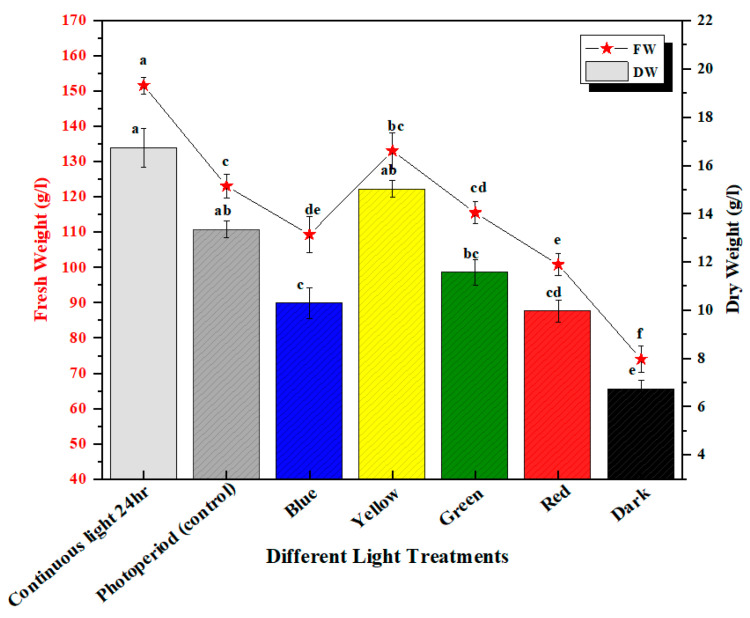
Fresh and dry weight produced under different monochromatic lights. The columns with similar letters are not statistically significant at *p* < 0.05.

In this study, the effect of a wide spectrum of monochromatic lights was analyzed for the enhancement of biomass productions in in vitro *M. oleifera* callus cultures. Callus cultures were optimized at 2.5 mg/L TDZ and 1.00 mg/L NAA growth hormone treatments (Supplementary Table S1) and placed in wide-spectrum monochromatic lights. The callus cultures placed under photoperiod conditions (16 h light/8 h dark) were recognized as a control in this experiment (Figure 1). The highest fresh weight production (FW: 133.07 g/L) was seen in continuous white light (24 h) followed by yellow light (FW: 133.07 g/L), as compared to the control (FW:112.05 g/L), and the lowest were seen in callus cultures placed under continuous dark (FW: 64.03 g/L) (Figure 2), whereas the highest dry weight (DW: 16.7 g/L) was observed in continuous white light (24 h) followed by yellow light (DW: 15.02 g/L), with respect to control (photoperiod 16 h light/8 h dark). The higher production of biomass in yellow light as compared to photoperiod (control) may be due to the better efficiency of photosynthetic systems and higher accumulation of bioactive compounds and flavonoids produced in callus cultures placed under yellow light. Moreover, the net photosynthesis value (Pn) under yellow light treatment was evidently better than the photoperiod [30] (Figure 2). Light signaling pathways regulate the growth, differentiation, and metabolism in callus cultures. Different light wavelengths deferentially evoke different responses for the enhanced production of biomass in the in vitro callus cultures [3,31,32]. The highest biomass under continuous white light was due to the excess level of energy that has a major role in plants’ physiological processes, morphogenesis, and biochemical pathways, including the biosynthesis of primary and secondary metabolites. Similar results were observed in cell cultures of *Withania somnifera* exposed to continuous white light for the high accumulation of biomass [33]. It was also observed from the previous studies for the high biomass accumulation for *Artemisia absinthium L.* callus culture from continuous white light with respect to other lights as well as in a callus culture of *Lepidium sativum L.* placed under continuous white light (24 h) [31,34]. The differential spectral light influence changes significantly with the intensity and quality of applied lights, time of exposure, and nature of plant species [32].

### 2.2. Accumulation of Phenolic and Flavonoid Contents

**Figure 3:**
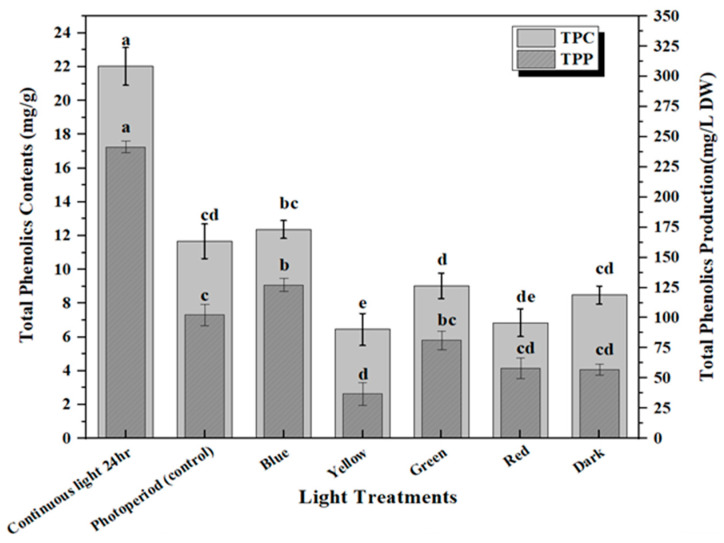
Differential effect of lights on TPC (mg/g DW) and TPP (mg/L) accumulation in in vitro callus cultures. The columns with similar letters are not statistically significant at *p* < 0.05.

**Figure 4:**
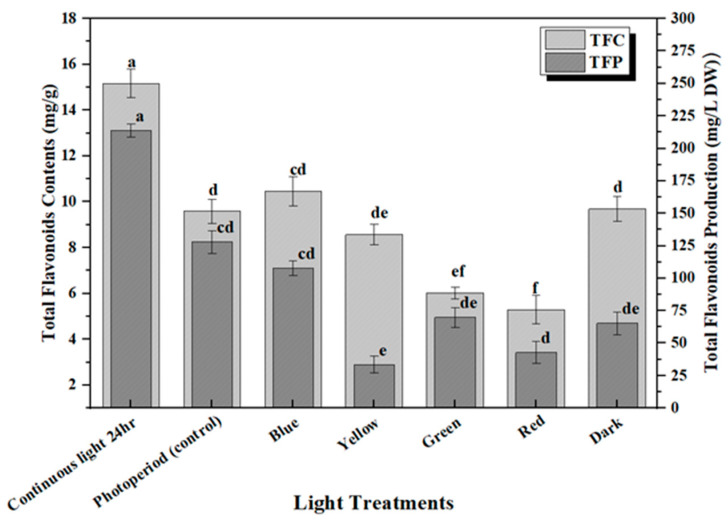
Differential effect of lights on TFC (mg/g DW) and TFP (mg/L) accumulation in in vitro callus cultures. The columns with similar letters are not statistically significant at *p* < 0.05.

The production of secondary metabolites in plants protects them against stress either environmentally or pathogenically through the regulation of plant growth and developmental processes [35]. Flavonoids are the major group of bioactive constituents present in the plants that control various physiological responses under stress conditions. The effective influence of multi-spectral lights on the optimum productions of plant secondary metabolites in the callus culture of *M. oleifera* has been investigated in this study. Highest accumulation of total phenolic content (TPC: 18.03 mg/g) and phenolic production (TPP: 287.5 mg/L) was noted in the callus culture grown in continuous white light (24 h), followed by blue light (TPC: 12.37 mg/g and TPP: 124.07 mg/L), and dark (TPC: 8.49 mg/g and TPP: 183.5 mg/L) (Figure 3). The increased production of TPC and TPP accumulation in the in vitro callus cultures under blue light compared to photoperiod is due to an increase in the transformation efficacy of secondary metabolites, which can possibly lead to the diversions in some of the chain reactions of biosynthetic pathways. Similar results were shown for total flavonoid content in response to continuous white light for 24 h (TFC: 15.2 mg/g and TFP: 212.75 mg/L) followed by blue light (TFC: 11.05 mg/g and TFP: 108 mg/L) and dark (TFC: 9.01 mg/g and TFP: 64.03 mg/L) compared to the control (Figure 4). There is a positive trend in biomass production grown under different lights (Figure 2) with total phenolic content (TPC) (Figure 3) and total flavonoid content (TFC) (Figure 4). Continuous white light for 24 h may upregulate the transcription levels of flavonoid biosynthetic genes to promote flavonoid accumulation. Light elicitation is among the best approach for the biosynthesis and enhancement of the important metabolites and nutraceuticals [32,36]. The variation in the total flavonoid production may be attributed to the activation of key enzyme phenylalanine ammonia-lyase (PAL), which is involved in flavonoid biosynthesis. Enhanced anthocyanin production was noted in hairy root cultures of *Echinacea purpurea* placed in continuous white light and anti-cancer compounds in a callus culture of *Fagonia indica*, as well as increased production of phytochemicals in adventitious root cultures of *W. somnifera* L. as reported in previous studies [3,37,38,39]. Contrarily reports have also shown that blue and red light have a significant effect on increasing secondary metabolites in in vitro callus cultures [32,40,41,42,43,44]. Therefore, it is evident from the aforementioned reports that there is a strong effect of light quality and intensity on the enhancement of commercially viable bioactive compounds that varies from plant species to species.

### 2.3. HPLC-Based Metabolite Quantificaton

Plants produce a large array of phytochemicals that could play significant roles in survival and growth, along with defending them against intruding pathogens. Biosynthesis of such commercially viable, pharmaceutically important metabolites can be enhanced through elicitors, especially through light elicitation. The quantifications of such metabolites are mostly determined by HPLC (High-performance liquid chromatography). *Moringa oleifera* contains a multitude of important phytochemicals. In this study, the quantification of the important metabolites quercetin, chlorogenic acid, neochlorogenic acid, kaempferol, and apigenin produced under the influence of wide-spectrum, monochromatic lights have been performed through HPLC (High-performance liquid chromatography) (Table 1). Under the strong influences of white light for 24 h, the maximum level of metabolites was produced. All such compounds were recorded as being highest in continuous white light (24 h). The same results were reported in an in vitro callus culture of *F. indica* in continuous white light by Nadeem et al. as well as in a callus culture of *Ocimum basilicum* under white light by Khan et al. [3,45]. Contrarily, the effect of red light for enhanced production of silymarin in a callus culture of *Silybum marianum* was reported by Younas et al. [40], and the effect of red light on the enhanced production of cucurbitacin in in vitro cultures was reported by Kuo et al. [46]. The function of these secondary metabolites as anticancer, neuroprotective, and antioxidant is to minimize the oxidative stress produced in the cell [47]. The type, dose, nature, and exposure time of the light are required for provoking a strong elicitation effect for the enhanced production of commercially important metabolites in in vitro callus cultures [48]. The pharmaceutical industry highly values these compounds. Different reports have shown that they play a critical role in neuroprotection and cancer prevention by reducing oncogene expression and oxidative stress, cell death, and cell cycle inhibition [47]. By interacting with cytochrome p450 and other important enzymes, polyphenolic substances can improve their anticancer potential [49].

### 2.4. Free Radical Scavenging Activity

**Figure 5:**
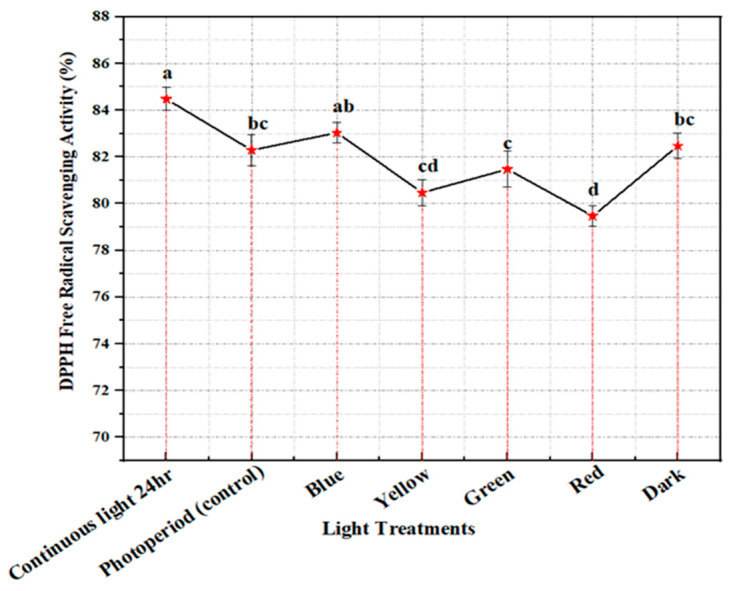
DPPH free radical scavenging activity of in vitro callus cultures of *M. oleifera* under light treatments. The columns with similar letters are not statistically significant at *p* < 0.05.

DPPH (2,2-Diphenyl-1-picrylhydrazyl) is a stable organic, paramagnetic free radical with a strong absorption band at 517 nm. At room temperature, it produces a purple solution in methanol. It loses its free radical property by accepting an electron or a free radical species to become a stable diamagnetic molecule. As a result, it changes its color from purple to yellow. This method is easy, and sensitive so that it can rapidly scrutinize the free radical scavenging capacity of antioxidants present in the sample. Large quantities of reactive oxygen species (ROS) are accumulated in plants under stressful conditions that have dangerous effects on the development, growth, and the destruction of cells, especially via DNA degradation [50,51]. Plants produce antioxidant enzymes against ROS because they have a natural ability for defending mechanisms to minimize the deleterious effects of stress [52]. The production of flavonoids, phenolics, terpenoids, and carotenoids by plants acts as antioxidant compounds which can provide protection against harmful ROS and RNS species [53,54]. In the current study, in vitro callus cultures of *M. oleifera* (PGR optimized) were placed under the differential spectral lights for 4 weeks and it was observed that the maximum antioxidant activity of 84% produced calli placed under continuous white light for 24 h, followed by blue light (81.7%), and dark (79.67%), with the lowest DPPH value of 59.58% in yellow light (Figure 5). An apparent trend can be seen between the production of phytochemicals (flavonoids and phenolics) (Figure 3) and free radical scavenging under the influence of continuous white light for 24 h. Similar results were noted by Khan et al. in free radical scavenging activity and increased production of phenolics and flavonoids under the influence of continuous white light in in vitro callus cultures [45]. Our results are in accordance with studies by Ali et al. and Beckwith et al. in which continuous light enhanced free radical scavenging activity due to the enhanced production of catalase enzymes [31,55].

### 2.5. Multispectral Light Effects on the Antioxidant Potential of the Callus Culture of M. oleifera

**Figure 6:**
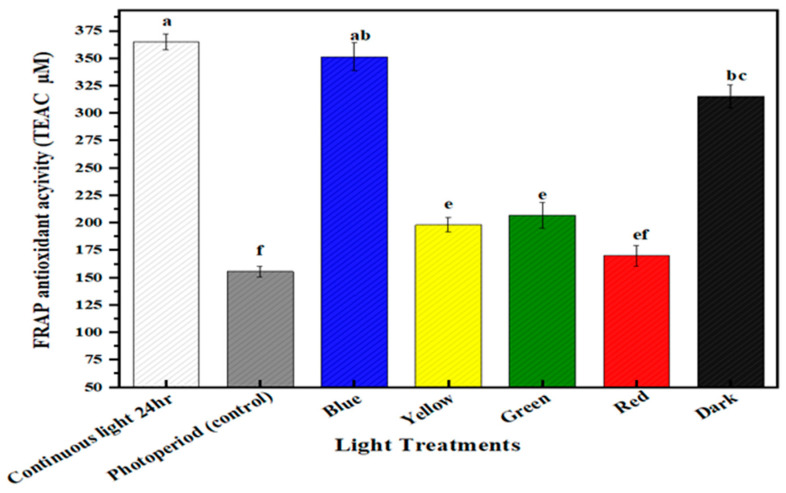
In vitro antioxidant FRAP activities of callus culture of *M. oleifera* under different light treatment. The columns with similar letters are not statistically significant at *p* < 0.05.

**Figure 7:**
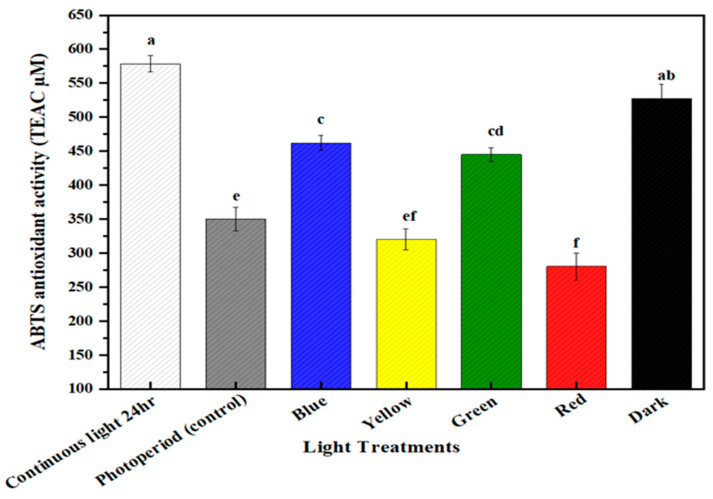
In vitro antioxidant ABTS activities of a callus culture of *M. oleifera* under different light treatments. The columns with similar letters are not statistically significant at *p* < 0.05.

The antioxidant values of in vitro callus cultures of *M. oleifera* grown under differential spectral lights were calculated through FRAP and ABTS assay. ABTS (2,2′-azino-bis (3-ethylbenzothiazoline-6-sulphonic acid)) assay is a radical cation decolorization method used to determine the antioxidant potential of samples. It works on the principle of the hydrogen atom transfer (HAT) mechanism. When ABTS is incubated with Na₂S₂O₈, it forms an ABTS cation (ABTS•+) that is deep blue in color and is highly reactive towards antioxidants. When mixed with a hydrogen-donating antioxidant, it was rapidly decolorized by accepting an electron pair and became nonradical. FRAP assay is another important indicator to evaluate the antioxidant capacity of the sample that is mainly based on reducing the capacity of Fe^3+^ to Fe^2+^ conversion (electron transfer mechanism). On reduction, it forms a blue-colored complex of Fe^+2^. This assay is used to ascertain the antioxidant potential of phenolic compounds present in the sample. The in vitro callus cultures of *M. oleifera* under continuous white light for 24 h produced the highest value of FRAP (365.37 TAEC uM), followed by blue light (350 TAEC uM), and dark (315.03 TAEC uM) (Figure 6). Similarly, ABTS antioxidant activity was recorded as being highest in continuous white light for 24 h, (550 TAEC uM), followed by dark (520 TAEC uM), and blue (467.78 TAEC uM) (Figure 7). Both antioxidant potential assays were measured in TEAC. When exposed to multispectral lights, there was a significant association between antioxidant potential and secondary metabolite accumulation. Under the influence of continuous white light for 24 h, the enhanced productions of phytochemicals (phenolics and flavonoids) could possibly be the reason for the enhanced antioxidant activity of in vitro callus cultures. The apparent trend has previously been reported between secondary metabolite production and antioxidant activity [56,57].

## 3. Material and Methods

### 3.1. In Vitro Seed Germination

*M. oleifera* seeds were collected from PCCL (plant cell and tissue culture laboratory) seed bank at Quaid-i-Azam University, Islamabad, Pakistan. Seeds were thoroughly washed with distilled water and were set to dry naturally in shade. Seed viability was determined by conducting a water flow test. Sterile seeds were first sterilized for one minute in HgCl_2_ solution (0.1%), followed by a 30-s wash in distilled water and 70% ethanol solution. Afterward, seeds were inoculated in a MS0 (Murshige and Skoog) medium, with an addition of 3% sucrose and 0.8% agar. The pH of the medium was adjusted at 5.65–5.75 and then sterilized with an autoclave at 121 °C for 20 min before inoculation. Inoculated seeds were placed in growth-specific chambers at 25 ± 1 °C and 16/8 light/dark cycles for 28 days.

### 3.2. In Vitro Establishment of Callus Culture

Twenty-eight-day-old in-vitro-derived plant leaves were utilized for an explant source. Leaf explants (4–5, 0.5 cm^2^) were excised using an autoclaved surgical blade and inoculated in MS media additionally added with optimized plant growth regulators, α-naphthalene acetic acid (NAA, 1.00 mg/L) + thidiazuron (TDZ, 2.50 mg/L), for callogenesis. Inoculated flasks were placed in a photoperiod cycle (16 h light/8 h dark) at 25 ± 1 °C temperature for 5 weeks. After the fifth week, the obtained callus was further subcultured onto freshly prepared growth media until a sizeable calli mass was obtained for light treatments.

### 3.3. Callus Exposure to Differential Lights

Subcultured fresh callus (1.0 g) was inoculated in fresh media and exposed to multi-spectral light treatments: control: Photoperiod (400–700 nm, 16/8 h light/dark), White Light (400–700 nm, 24 h), Dark (24 h), Yellow LEDs (570 nm, 24 h), Red LEDs (660 nm, 24 h), Green LEDs (510 nm, 24 h), and Blue LEDs (460 nm, 24 h) with the light of the intensity of 40–50 μMol/m/s (measured using Lux meter—SU10, Jeio-tech). The whole experiment was performed thrice.

### 3.4. Harvesting and Extraction of Callus Culture Samples

The callus culture of *M. oleifera* after 28 days of continuous exposures to different light treatments was dried on Whatman filter paper and gently pressed to remove excess moisture. Afterward, callus cultures were weighed to determine the fresh weight (FW) and were later placed in a 45 °C incubator for 24 h to determine dry weight. The dry callus was ground to a fine powder and analyzed for antioxidant and phytochemical content using the extraction protocol of Zahir et al. [58]. Methanol (0.5 mL) was added to the 0.1 g of dried callus sample and homogenized in a sonicator for 30 min along with 15 min of vortex. The cycle was repeated two times, followed by centrifugation at 15,000 rpm for 10 min. The supernatant was removed and stored at 5 °C.

### 3.5. Phytochemical Analysis

#### 3.5.1. Phenolic and Flavonoid Contents

To check the overall contents of phenolics produced in the callus culture of *M. oleifera*, Singleton and Rossi 28 method was employed using Folin–Ciocalteu (FC) reagent. Callus extract (20 µL), Na_2_CO_3_ solution (90 µL), and FC reagent (90 µL) were mixed and placed in a 96-well microplate. At 630 nm, a microplate reader (Thermo Scientific Multiskan GO) was used for measuring the absorbance of the samples after the incubation for 5 min. The standard used was Gallic acid as GAE/g of gallic acids. The following formula was used to calculate the production of phenolics:

Total phenolic production (mg/L) = DW (g/L) × TPC (mg/g)(1)

The total flavonoid content was calculated by Abbasi et al., method of Aluminum chloride colorimetric with slight modifications [3]. Methanol extract of callus (20 µL), aluminium chloride (10 µL), potassium-acetate (10 µL), and dH2O (160 µL) were mixed and placed in 96-well microplate with an incubation time of 30 min. The absorbance was calculated using microplate readers (Thermo Scientific Multiskan GO) at 415 nm wavelength. The flavonoid production was measured using the following formula along with quercetin as standard (QE/g).

Total flavonoid production (mg/L) = DW (g/L) × TFC (mg/g)(2)

#### 3.5.2. HPLC Quantification of Metabolites

The production of commercially viable phytochemicals in in-vitro-derived callus cultures of *M. oleifera* was quantified by HPLC according to Khurshid et al. [59], equipped with a photodiode array detector, and a pump from Varians-Prostar 230, Metachim Degaset, autosampler Varians- Prostar 410, for separation and quantification, and a Purospher (Merck) RP-18 column was used for separations. Two solvents (A: acetonitrite and B: formic acid acidified (0.1 percent *v*/*v*) ultra-pure water) were used in the HPLC system’s mobile phase. During the 60-min run, the mobile phase composition ranged from 5:96–100:0 (solvent A:B, *v*/*v*), with linear gradients with 0.80 mL/min flow rates. After every single run, a 10-min re-equilibration period was used. At 204 nm, *M. oleifera* compounds were detected and quantified based on retention times compared with commercially available standards, and all the results were expressed as µg/g DW.

### 3.6. Ferric-Reducing Antioxidant Power (FRAP) Assay

The ferric-reducing potential was calculated by Benzie and Strain protocol with slight modification [60]. At room temperature, 10 µL of the callus extract sample was mixed with 190 µL of FeCl_3_. Next, 6H2O, 300 mM acetate buffer pH 3.6, and 10 mM TTPTZ was mixed in 1:10:1 (*v*/*v*) reagent and stored for 15 min. A microplate reader was utilized to note the sample absorbances at 630 nm wavelength, and the results were expressed as TEAC (Trolox C equivalent antioxidant capacity).

### 3.7. Antioxidant ABTS Assay

ABTS reagent, as studied by Tagliazucchi et al. [61] was used for the measurement of an antioxidant’s potential for the callus extract. ABTS solution was made using 2.45 mM potassium persulphate mixed with 7 mM 2,2-azinobis-3-ethylbenzthiazoline- 6-sulphonic acid salt at a 1:1 ratio and incubated at 25 ± 2 °C in the dark conditions for 16 h. The sample extract was added at 25 °C, and a microplate-reader was utilized to measure absorbance at 734 nm; the results were expressed as TEAC.

### 3.8. Statistical Analysis

The experiments were replicated three times and repeated twice for accuracy. All of the experiment treatments were executed under the same environmental conditions. Mean values and standard errors were calculated for each experiment. Graphs were made in Origin pro-2018. The information was shown as a mean with a standard deviation indicated. Analysis of variance (ANOVA) was used for the statistical analysis of the experimental data and means were separated using HSD Tukey test *p* < 0.05.

## 4. Conclusions

In conclusion, the effect of wide-spectrum monochromatic lights was investigated in in vitro callus cultures of *M. oleifera*. The calli placed under different lights for 28 days were harvested and analyses of biomass, synthesis of phytochemicals, and biological activities were performed. The highest dry weight accumulation was noted in callus cultures treated with continuous white light for 24 h. Furthermore, continuous white light triggered the enhanced production of secondary metabolites, flavonoids, and phenolics biosynthesis in in vitro callus cultures of *M. oleifera*. The quantification of phytochemicals with HPLC showed the maximum accumulation of apigenin, luteolin, neochlorogenic acid, p-coumaric acid, chlorogenic acid, kaempferol, and quercetin in in vitro callus cultures of *M. oleifera* kept under continuous white light. Similarly, the highest antioxidant activities were also observed in calli placed under continuous white light. There was a positive correlation between the biosynthesis of phytochemicals and the biological activities in in vitro callus cultures of *M.oleifera*. This study shows the effect of wide spectrum lights on the sustainable production of nutraceuticals and phytochemicals.

## Figures and Tables

**Table 1:** The effect of multispectral light on the production of polyphenolic metabolites in an *M. oleifera* callus culture. Means and standard errors from triplicates are represented. The bold values indicate highest metabolite accumulation.

Marker Compounds (µg/g DW)	Different Lights Treatment						
	Control	Red	Green	Blue	Dark	White Light	Yellow
Apigenin	594.65 ± 0.009	558.09 ± 0.03	574.63 ± 0.21	611.50 ± 0.51	627.60 ± 0.07	**751.19 ± 0.44**	459.56 ± 0.011
Luteolin	751.19 ± 0.004	592.29 ± 0.05	715.88 ± 0.33	540.40 ± 0.28	0.169 ± 0.016	**798.29 ± 0.75**	433.39 ± 0.077
p-coumaric acid	89.07 ± 0.006	79.08 ± 0.07	148.41 ± 0.05	105.43 ± 0.047	137.09 ± 0.63	**159.03 ± 0.54**	78.07 ± 0.086
Neochlorogenic acid	789.65 ± 0.064	611.16 ± 0.02	635.08 ± 0.19	826.75 ± 0.044	754.19 ± 0.65	**998.38 ± 0.25**	662.91 ± 0.006
Chlorogenic acid	144.92 ± 0.855	128.63 ± 0.82	86.93 ± 1.209	**188.14 ± 1.55**	138.07 ± 1.66	**210.57 ± 0.99**	144.92 ± 0.66
Quercetin	592.29 ± 1.776	701.07 ± 2.05	665.56 ± 1.99	721.45 ± 2.44	821.82 ± 3.055	**959.92 ± 1.87**	433.39 ± 1.363
Kaempferol	468.70 ± 1.055	537.87 ± 0.79	565.89 ± 0.622	721.65 ± 0.83	844.51 ± 1.22	**1016.04 ± 1.22**	405.97 ± 0.92

## Data Availability

All of the data are included in the present study and the online associated Supplementary Materials.

## Supplementary Materials

The following supporting information can be downloaded at: https://www.mdpi.com/article/10.3390/molecules28031497/s1, Table S1: PGRs concentrations, callus initiation (day), fresh weight (g/L), Dry weight (g/L), morphology of calli, formed from leaf explants under Photoperiod light (16-h light and 8-h dark).

## Sample Availability

Samples of the compounds are available from the authors upon request.
