# Supplementary material for: Effect of Wide-Spectrum Monochromatic Lights on Growth, Phytochemistry, Nutraceuticals, and Antioxidant Potential of In Vitro Callus Cultures of Moringa oleifera
Source: Molecules. 2023 Feb 3;28(3):1497. doi: 10.3390/molecules28031497 (PMC9921732; doi:10.3390/molecules28031497)
Supplement: Supplementary file 1 [file molecules-28-01497-s001.zip › molecules-2077529-supplementary.pdf]

Supplementary Table S1: PGRs concentrations, callus initiation (day), fresh weight (g/L), Dry weight (g/L), morphology of calli, formed from leaf explants under Photoperiod light (16-h light and 8-h dark).

S. no.	PGRs treatments (mg/L)	Callus initiation (day)	Callus color	Callus texture	Fresh Weight g/L	Dry weight g/L
1	NAA 0.1	17th day	Y	C	38.07	4.92
2	NAA 1.0	15th day	YG	C	43.23	5.76
3	NAA 2.5	14th day	YB	C	59.55	6.68
4	NAA 5.0	14th day	YB	C	83.37	8.25
5	NAA 10	14th day	YB	F	73.91	7.54
6	TDZ 0.1	15th day	YB	F	47.56	6.26
7	TDZ 1.0	14th day	YB	C	53.01	6.45
8	TDZ 2.5	14th day	YB	C	88.43	8.78
9	TDZ 5.0	14th day	B	C	74.82	7.61
10	TDZ 10	14th day	YB	C	64.64	7.32
11	NAA 1+TDZ 0.1	14th day	Y	C	45.98	5.83
12	NAA 1+TDZ 1.0	14th day	YB	C	89.54	8.82
**13**	**NAA 1+ TDZ 2.5**	**14th day**	**YB**	**F**	**114.98**	**12.78**
14	NAA 1+ TDZ 5.0	14th day	B	F	82.01	8.13
15	NAA 1+ TDZ 10	14th day	B	F	76.0	7.64
16	BAP 0.1	15th day	B	F	32.35	4.52
17	BAP 1.0	14th day	B	F	39.22	4.76
18	BAP 2.5	16th day	B	F	46.45	5.89
19	BAP 5.0	14th day	G	C	52.07	6.37
20	BAP 10	14th day	YB	C	48.98	6.18
21	NAA 1 + BAP 0.1	14th day	YB	C	44.76	5.81
22	NAA 1+ BAP 1.0	15th day	YB	C	57.64	6.78
23	NAA 1+ BAP 2.5	13th day	GB	C	83.67	8.46
24	NAA 1 + BAP 5.0	14th day	GB	C	62.83	7.18
25	NAA 1+ BAP 10.0	13th day	B	F	75.61	7.69

C = Compact; F = Friable; YG = Yellowish Green; YB= Yellowish Brown; GB= greenish brown ; Y = Yellow; B= Brown
